# The Effects of Processing Fluency in Prosocial Campaigns: Effort for Self-Benefit Produces Unpleasant Feelings

**DOI:** 10.3389/fpsyg.2020.01221

**Published:** 2020-06-18

**Authors:** Yaeeun Kim, Yaeri Kim

**Affiliations:** ^1^ Department of Marketing and Supply Chain Management, Fox School of Business, Temple University, Philadelphia, PA, United States; ^2^ Orfalea College of Business, California Polytechnic State University, San Luis Obispo, CA, United States; ^3^ Department of Marketing, College of Business Administration, Sejong University, Seoul, South Korea

**Keywords:** processing fluency, appeals, self-benefit, social benefit, prosocial campaign, unpleasant feelings

## Abstract

This study investigates how consumers’ intentions related to prosocial campaigns were accompanied by metacognitive experiences. Two studies examined how the relationship between appeal type (self-benefit vs. social benefit) and the level of processing fluency (easy vs. difficult) influenced attitudes toward prosocial campaigns. The findings revealed that individuals who were manipulated to find self-benefit appeal displayed less favorable attitudes toward disfluent prosocial campaigns than those who were manipulated to find social benefit appeal. The underlying mechanism of this result was that the extra effort invested to understand prosocial campaigns with difficult processing fluency produced unpleasant feelings.

## Introduction

Effective marketing campaigns have become more important than ever with the growth of digital marketing. Global spending on digital advertising is expected to be US$335 billion in 2020 (Statista, 2019). The recent surge of research related to digital marketing has greatly improved our understanding of how to effectively deliver messages to customers through digital marketing. Previous studies have, for example, addressed the effects of processing fluency of marketing campaigns (Labroo and Kim, 2009; Brakus et al., 2014). Another research stream has investigated the effects of appeal type on prosocial behavior (self-benefit vs. other-benefit; White and Peloza, 2009). However, there is a lack of research that explores the interaction effects between appeal types and campaign processing fluency. Thus, the current study aims to investigate the combined effect of the level of processing fluency and appeal type on prosocial campaigns. In particular, there are two different types of appeals to encourage consumer participation in marketing campaigns. Self-benefit appeals emphasize what participants for social campaigns can gain for themselves, while social benefit appeals stress the benefits to the people or organizations who receive benefits (White and Peloza, 2009). Processing fluency is generally separated into two levels (difficult-to-process and easy-to-process) based on how much effort is needed to process the information presented (Schwarz, 2004).

The current study also aims to determine what kind of advertising strategy increases overall consumer preference when the appeal type and the message of the advertisement are incongruent (e.g., when they motivate prosocial behavior by increasing self-benefit). More specifically, this study investigates how consumers’ thoughts and decisions about prosocial campaigns were accompanied by metacognitive experiences. Although self-benefit is a common motivation for prosocial behavior, people may struggle to find valid reasons to act out of self-interest or for monetary rewards. Thus, investing extra effort into a self-benefit appeal may be viewed as dishonorable (Zhang et al., 2010). To resolve this, we designed two studies examining how the relationship between appeal type and processing fluency influenced attitudes toward prosocial campaigns. To manipulate the level of processing fluency, two different formats are applied: conceptual fluency for Study 1 and perceptual fluency for Study 2. The findings were consistent, and they revealed that individuals who received a self-benefit appeal showed unfavorable attitudes, caused by unpleasant feelings, toward disfluent prosocial campaigns that required extra effort.

This research contributes to the literature on branding and advertisement by providing the underlying affective mechanisms of the effects on attitudes toward prosocial campaigns of the relationship between processing fluency and appeal type. For business practitioners, the findings also suggest that improving processing fluency may increase consumer participation in prosocial campaigns.

## Theoretical Background

### Appeal Type

Typically, business practitioners and charities use two different types of appeals to encourage consumer participation in marketing campaigns: self-benefit appeals and social benefit appeals. Self-benefit appeals focus on benefits for the donor, while social benefit appeals focus on benefits for the people or organizations receiving benefits (White and Peloza, 2009). Previous work on prosocial behavior was focused primarily on the effects of altruism in increasing the motivation to engage in prosocial behavior. However, researchers have proved that individuals can also be motivated to participate in prosocial campaigns by self-interest or monetary rewards (Ariely et al., 2009), particularly in private prosocial activities. Thus, the current study intends to explore the effects of the self-benefit appeal on consumer attitudes toward prosocial behavior.

### The Moderating Role of Processing Fluency

With appeal type as one variable, we made the level of processing fluency the other variable. Processing fluency refers to the ease or difficulty with which information can be processed (Schwarz, 2004). More specifically, conceptual fluency refers to semantic relatedness or conceptually based knowledge (Shapiro, 1999). For example, Whittlesea (1993) reveals that semantic processing can lead to conceptual fluency by manipulating the place of the target word (e.g., boat) either in the predictable (e.g., “The stormy seas tossed the boat”) or unpredictable place (e.g., “He saved his money and the boat”). Perceptual fluency refers to the ease of identifying the perceptual features of a stimulus, such as its form, size, or visual details (Jia et al., 2016; Graf et al., 2017). The level of processing fluency in a campaign can be manipulated, for example, by the sharpness of text and images (Labroo and Kim, 2009).

Even though processing fluency can be represented with different forms of conceptual and perceptual fluency, recent research on processing fluency has proven that the independent form of fluency exerts the same influence on consumer judgments (Brakus et al., 2014). However, the level of processing fluency was revealed to have contradictory effects on attitudes. Easy-to-process stimuli are more positively evaluated (Reber et al., 2004; Brakus et al., 2014) through a feelings-as-information model. For example, conceptual fluency increases favorable attitudes (Schwarz, 2004), specifically in the context of brand evaluations and customer-based brand equity (Sirianni et al., 2013; Dogerlioglu-Demir et al., 2017). Context congruity increases conceptual fluency, which allows the memory of information to be more accessible (Lee and Labroo, 2004). Clear images, line drawings, and pictures increase perceptual fluency because subjective ease is viewed positively (Reber et al., 2004; Schwarz, 2004). Managerially, easy processing helps consumers to relate immediately to a brand, is more conducive to positive advertising through branding (An et al., 2019), and increases store revisit intention (Murray et al., 2019) and recognition and understanding of a brand logo (Kim and Lim, 2019). As such, processing fluency was predicted to have a positive effect on consumer attitudes. Although previous research has discovered the effectiveness of easy-to-process text and images, the underlying mechanism of processing fluency has not yet been emphasized (Carrillat et al., 2015; Park and Ryu, 2019).

Other research found that difficult to process, rather than ease to process, improves the evaluation toward the target object. The more effort that people put into a pursuit, the more they value it (Festinger, 1962). Greater efforts invested to understand the information presented (e.g., consuming energy or spending time to read the advertisement) increase the valuation of the requested action (e.g., volunteering or donation), especially when that action is regarded as a reward for successful performance (Norton et al., 2012). This effect can be interpreted based on naïve theories reflecting how effortful processing is considered more desirable by perceiving the means as instrumental to achieving an outcome (Deval et al., 2012). Effort investment theory also postulates that people assume difficult tasks require extra effort because they result in greater rewards (Klein et al., 2005). Because there is a strong association between sacrifice and altruism (Lin-Healy and Small, 2013), people prefer prosocial behavior that is accompanied by effort and pain (Olivola and Shafir, 2013). However, the joint effects of the level of processing fluency and appeal type on prosocial campaigns are unclear. This will be discussed in the next section.

### Affective Response Within the Interaction Effect of Appeal Type and Processing Fluency

Metacognitive feelings are influential in judging the instrumentality of the means to accomplish one’s goal and comprehending one’s evaluation of a target object (Labroo and Kim, 2009; Ishikawa et al., 2019). Previous work on prosocial behavior has focused primarily on positive emotions as the underlying mechanisms affecting participation in prosocial activities. For example, the “warm glow” as a positive emotional response to prosocial behavior (Andreoni, 1990, 1995) increased participant satisfaction with sustainable marketing programs (Giebelhausen et al., 2016) and increased purchase intentions toward green energy brands (Hartmann and Apaolaza-Ibáñez, 2012). In addition to the effects of positive emotions, other research has demonstrated the prosocial behavioral consequences of negative emotions (Brief and Motowidlo, 1986; Van Doorn et al., 2014). An unpleasant feeling (Baumeister et al., 1994) stems from self-evaluation or social interaction and is related to moral standards (Kugler and Jones, 1992). As such, unpleasant emotions can prompt people to perform good deeds in order to eliminate those feelings (Xu et al., 2012, 2014). In some other cases, unpleasant feeling appeals decreased purchase intention in the context of cause-related marketing campaigns (Singh et al., 2017).

To reconcile the conflicting findings, this study aims to investigate how the negative affective response works as a booster or suppressor for prosocial behavior in the context of processing fluency. For example, Labroo and Kim (2009) studied the interacting effects of goal type and processing fluency on consumer decision making. When the desired state (e.g., fitness is a virtue) conflicts with a temptation (e.g., chocolate consumption), participants presented less favorable attitudes toward the chocolate advertisement with difficult-to-process stimuli. Consistently, Schwarz (2004) revealed that difficult-to-process stimuli were evaluated less positively when the target object is not considered as a means to achieve a goal. More fundamentally, the well-documented cognitive depletion theory can explain this difficult-to-process reluctance effect (Pearson et al., 2013). Existing conflicts between the desired state (self-benefit appeal) and a current state of behavior (processing prosocial campaign) may lead to cognitive depletion and lead people to choose stimuli by putting in less effort. In other words, difficult-to-process stimuli, which require extra effort, should be viewed less positively.

Regarding the affective response, we expect that people may experience unpleasant emotions when they are forced to process disfluent stimuli and when self-benefit appeals are manipulated. Merely thinking about the selfish benefits of participating in the prosocial campaign may not increase unpleasant feelings (Peloza et al., 2013). However, we expect that effortful information processing negatively influences individual responses. We can find a basis for this assumption from the research on congruity, which also suggests that individual responses are sensitive to the degree of congruence between personal values and the appeal type in advertisements (Dens et al., 2017). Advertisements that require extra effort to process are expected to increase unpleasant emotions, resulting in a decrease in prosocial motivations. Findings in the previous research suggest that unpleasant feelings are linked with unjustifiable efforts in processing fluency (Kivetz and Simonson, 2002; Labroo and Kim, 2009). However, this assumption was not supported by empirical results. Therefore, the current research examines the underlying affective response as an explanation for consumers’ attitudes toward disfluent advertisements.

In this study, we predict that a self-benefit appeal would not increase participants’ unpleasant feelings toward easy-to-process stimuli. Existing research on prosocial behavior revealed that enhanced fluency originates from the congruence between individually pursuing values and messages from advertisements, thus promoting engagement in prosocial behavior (Kidwell et al., 2013). Therefore, we expect that the appeal types would not change the attitudes toward campaigns when they are easy to process. Similarly, we expect that the appeal types do not induce unpleasant feelings toward campaigns when they are easy to process.

**Figure 1:**
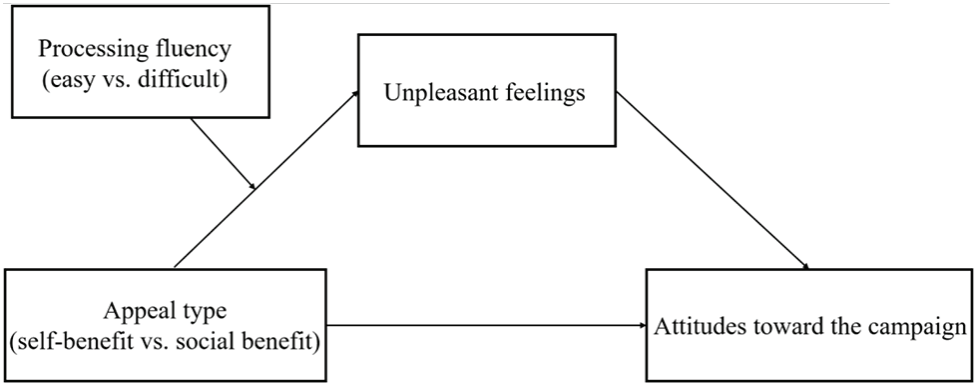
The conceptual framework.

Based on the thorough literature review above, we propose the following hypotheses (see Figure 1).

*Hypothesis* 1: the difficult-to-process campaign will increase participants’ unpleasant feelings in the self-benefit appeal condition more than in the social benefit appeal condition. However, the appeal type will not affect the participants’ unpleasant feelings in the easy-to-process campaign.
*Hypothesis* 2: the difficult-to-process campaign will decrease positive participant attitudes toward the campaign more in the self-benefit appeal condition than in the social benefit appeal condition. However, the appeal type will not affect participant attitudes toward the easy-to-process campaign.
*Hypothesis* 3: unpleasant feelings will mediate the interaction effect between the level of processing fluency and appeal type on participant attitudes toward the campaign.

## Materials and Methods

### Study Overview

Two studies tested our hypotheses in the context of prosocial campaigns. Study 1 examined how conceptual fluency interacted with appeal type in a prosocial campaign, while Study 2 examined a similar marketing campaign with perceptual fluency. Study 1 and Study 2 received the Institutional Review Board approval. Taken together, these experiments investigated how processing fluency affected motivation toward prosocial behavior and examined the affective response underlying the effects of appeal type on participation in prosocial campaigns.

### Study 1

The primary objective of Study 1 was to examine how the appeal types influenced consumers’ affective responses based on the level of conceptual fluency. The authors predicted that, in the self-benefit appeal condition, consumers would experience increased unpleasant feelings while processing a difficult-to-understand message with a lot of medical jargon because the participants would not be able to justify investing the extra effort. The authors also predicted that the participants would not show different affective responses to the easy-to-process campaign.

#### Procedure

Using Amazon Mechanical Turk, 311 participants in the United States (55.6% females; *M*_age_ = 37.35 years) were randomly assigned to one of four conditions in a 2 (appeal: self-benefit vs. social benefit) × 2 (processing: easy vs. difficult) between-subjects design. They were instructed that the objective of this study was to determine consumer behaviors regarding prosocial campaigns. First, the participants were exposed to either the social benefit or the self-benefit condition for an advertisement requesting volunteers for a local charity, the American Heart Association (White and Peloza, 2009). The social benefit condition highlighted two altruistic benefits: “Volunteering can generate well-being for people and make a difference in the community” and “This work is a good way to make a contribution and to benefit other people.” On the other hand, the self-benefit condition highlighted two personal benefits: “Volunteering can help to develop some new skills for your resume as well as to make some valuable contacts” and “You can take advantage of this activity to develop job skills and social networking” (Clary et al., 1998). Then, the participants were presented with campaigns that were either easy or difficult to understand (Zhang and Mattila, 2015). To manipulate the level of content processing fluency, the participants were asked to read a message from the American Heart Association that was either composed of scientific jargon or written in simple words. Specifically, in the difficult-to-process condition, participants read statements such as: “There are various types of cardiovascular disease, such as cor pulmonale, cardiac dysrhythmias, and cardiomyopathy. In general, the causes of cardiovascular disease are diverse, but atherosclerosis and/or hypertension are the most common.” In contrast, the participants in the easy-to-process condition read: “There are different types of heart disease, such as heart failure, abnormalities of the heart’s rhythm, and diseases of cardiac muscle. In general, heart disease refers to conditions that involve narrowed or blocked blood vessels, which can lead to a heart attack, chest pain, or stroke.”

After reading the scenarios, we asked participants to answer the manipulation check questions of appeal types with four items (*α* = 0.913) on seven-point scales (1 = strongly disagree, 7 = strongly agree; White and Peloza, 2009): “To what degree is this an altruistic appeal? (reverse coding)”; “To what degree is this appeal associated with looking out for the interests of others? (reverse coding)”; “To what degree is this an egoistic appeal?”; and “To what degree is this appeal associated with looking out for one’s own interests?” Then, unpleasant feelings experienced during the processing of the campaigns were measured with a seven-point scale (1 = not at all, 7 = very much): “The benefit addressed in the passage makes me feel guilty.”

### Results

#### Manipulation Check

A 2 × 2 ANOVA on the manipulation checks of the appeal conditions revealed that the participants interpreted the scenarios as the authors had intended. Participants assessed the self-benefit appeal as being more focused on self-benefits than the social benefit appeal (*M*_self-benefit_ = 4.96 vs. *M*_social benefit_ = 2.48; *F*(307) = 264.264; *p* < 0.0001). No other effects were significant (*F*s < 1).

#### Unpleasant Feelings While Processing the Campaigns

**Figure 2:**
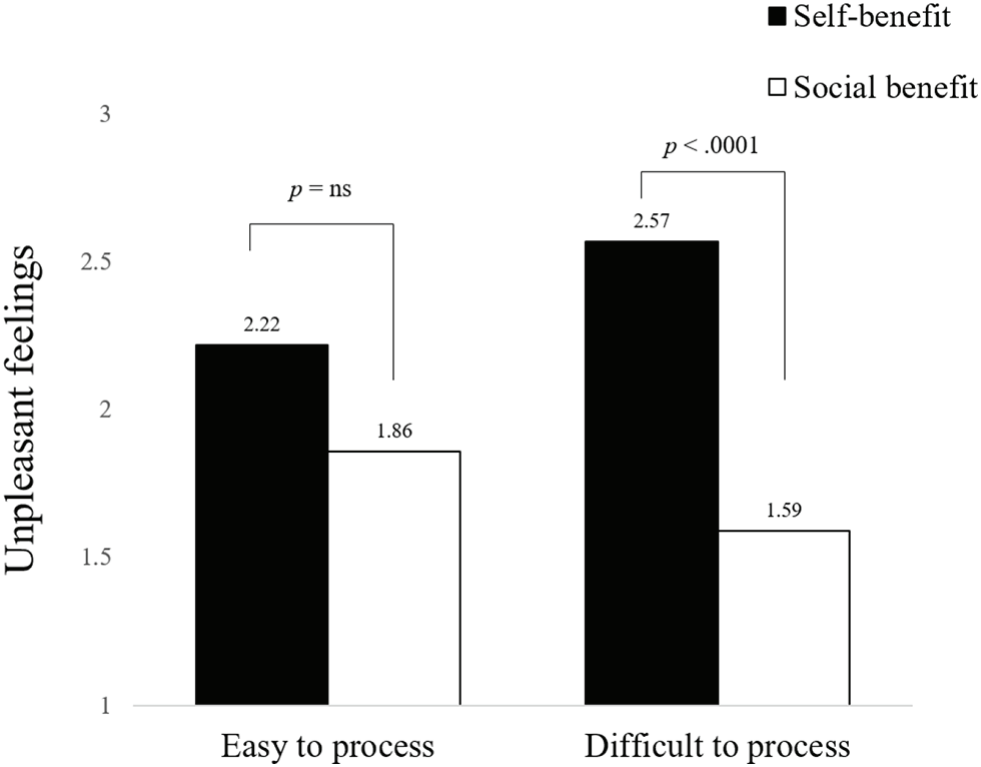
The moderating effect of appeal types and conceptual fluency on unpleasant feelings.

In addition, a 2 × 2 ANOVA on unpleasant feelings revealed a marginally significant interaction (*F*(1, 307) = 3.635, *p* = 0.058). More critically, as predicted in Hypothesis 1, the participants felt more unpleasant toward the difficult-to-process campaign when manipulated with the self-benefit appeal than with the social benefit appeal (*M*_self-benefit_ = 2.57 vs. *M*_social benefit_ = 1.59; *t*(307) = 4.283; *p* < 0.0001; see Figure 2). However, for the easy-to-process campaign, there was no difference in the unpleasant feelings experienced in the social benefit appeal condition and the self-benefit appeal condition (*M*_social benefit_ = 1.85 vs. *M*_self-benefit_ = 2.22; *t*(307) = 1.551; *p* = 0.122).

### Discussion of the Results

The results of Study 1 support Hypothesis 1 in that the difficult-to-process campaign increased participants’ unpleasant feelings in the self-benefit appeal condition more than in the social benefit appeal condition. These findings suggest that using the self-benefit appeal in prosocial settings can impair feelings toward the campaign, which can hamper the success of the campaign. However, as predicted, the easy-to-process campaign did not influence participants’ affective responses for either the self-benefit appeal or the social benefit appeal conditions.

### Study 2

In Study 1, we found that the difficult-to-process conceptual processing fluency makes individuals feel more unpleasant in the self-benefit appeal condition compared to the social benefit appeal condition. In addition to examining how appeal types and processing fluency affected unpleasant feelings toward the campaign, we also examined how appeal types and processing fluency affected attitudes toward the campaign. In this study, we replicated the results of Study 1 in the perceptual fluency context and examined the mediating role of unpleasant feelings to explain the effects on attitudes toward the campaign.

#### Procedure

**Figure 3:**
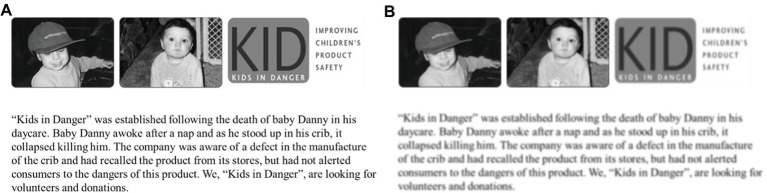
Stimuli of Study 2. **(A)** Easy-to-process campaign and **(B)** difficult-to-process campaign.

Using Amazon Mechanical Turk, 197 participants in the United States (50.3% females; *M*_age_ = 31.88 years) were randomly assigned to one of four conditions: 2 (appeal: self-benefit vs. social benefit) × 2 (processing: easy vs. difficult). The authors followed the same procedures as in Study 1; however, in Study 2, the level of processing fluency was manipulated by the sharpness of text and pictures in the campaign using the materials modified from Labroo and Kim (2009). For the difficult-to-process campaign in Study 2, the authors adjusted the sharpness by − 100% for the picture and − 45% for the text. For the easy-to-process campaign, the authors used an original clear picture and text (see Figure 3). After viewing an appeal scenario and a campaign picture, each participant answered all the measurements for manipulation checks, attitudes toward the prosocial campaign, unpleasant feelings while processing the campaign. The manipulation check questions of the appeal (*α* = 0.800) were measured as in Study 1. The participants then indicated their attitudes toward the campaign (*α* = 0.937) using six seven-point bipolar scales (bad/good, unfavorable/favorable, negative/positive, undesirable/desirable, harmful/beneficial, and unconvincing/convincing). Unpleasant feelings while processing the campaigns were also measured with a seven-point scale (1 = not at all, 7 = very much; “How much do you feel regret?”).

#### Results

##### Manipulation Check

A 2 × 2 ANOVA on the manipulation checks of the appeal conditions revealed that the participants interpreted the appeal scenarios as the authors had intended. Participants assessed the self-benefit appeal as being more focused on self-benefits than the social benefit appeal (*M*_self-benefit_ = 3.87 vs. *M*_social benefit_ = 2.72; *F*(193) = 34.199; *p* < 0.0001). No other effects were significant (*F*s < 1).

##### Attitudes Toward the Prosocial Campaign

**Figure 4:**
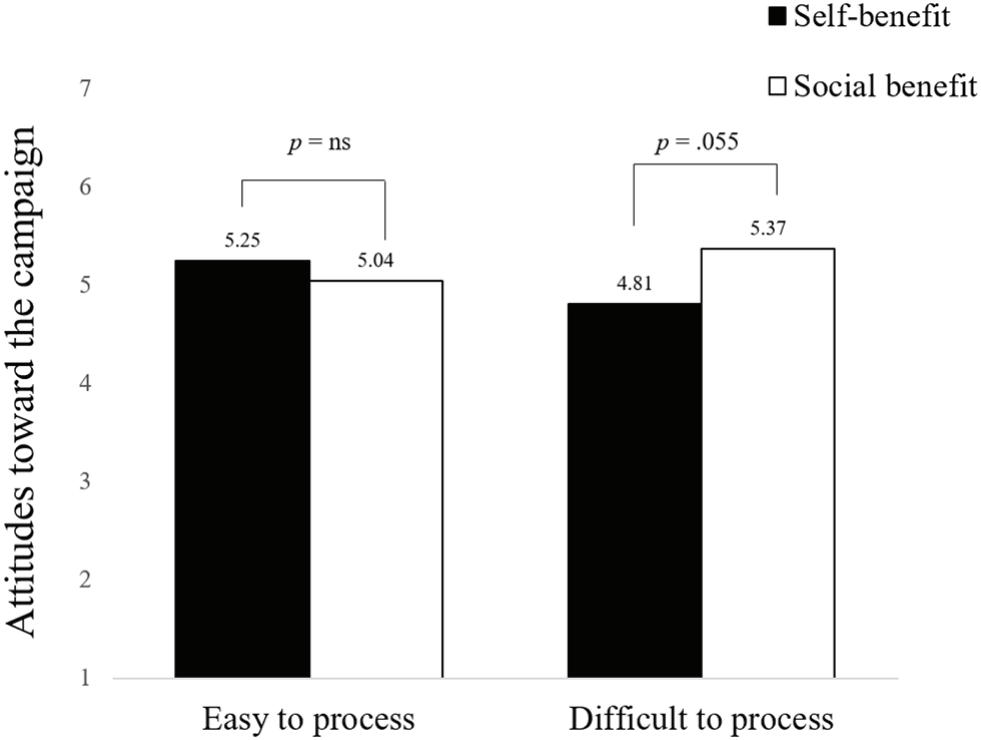
The moderating effect of appeal types and perceptual fluency on attitudes toward the campaign.

A 2 × 2 ANOVA on attitudes toward the prosocial campaign revealed a marginally significant interaction (*F*(1, 193) = 3.438, *p* = 0.065). As predicted in Hypothesis 2, planned contrasts showed that the participants rated attitudes toward the difficult-to-process condition with a blurred picture less favorably when manipulated with a self-benefit appeal than with a social benefit appeal (*M*_self-benefit_ = 4.81 vs. *M*_social benefit_ = 5.37; *t*(193) = 1.927; *p* = 0.055; see Figure 4). However, there was no difference in the easy-to-process condition (*M*_self-benefit_ = 5.25 vs. *M*_social benefit_ = 5.04; *t*(193) = 0.703; *p* = 0.483).

##### Moderated Mediation Analysis

Next, we conducted a moderated mediation test using appeal type as an independent variable (1 = self-benefit appeal, 0 = social benefit appeal), processing fluency as a moderator (1 = easy, 0 = difficult), unpleasant feelings as a mediator, and the attitudes toward the campaign as a dependent variable (Process Model 7; Hayes, 2017). A moderated mediation model revealed that unpleasant feelings mediated the interaction effect on attitudes [95% CI = (−0.3411, −0.0044)], supporting Hypothesis 3 (see Supplementary Table 1). The conditional indirect effect was only significant for the difficult-to-process campaign [indirect effect = −0.0754, *SE* = 0.0539; 95% CI = (−0.2309, −0.0022)]; it was not significant for the easy-to-process campaign [indirect effect = 0.0438, *SE* = 0.0450; 95% CI = (−0.0118, 0.1786)]. These analyses suggested that the participants had less positive attitudes toward the difficult-to-process campaign because of unpleasant feelings.

#### Discussion of the Results

The results of Study 1 were replicated in Study 2 for perceptual fluency. The authors also observed an increased negative affect in the difficult-to-process campaign, which influenced participant attitudes toward the campaign. The participants had less positive attitudes toward the difficult-to-process campaign in the self-benefit appeal condition than in the social benefit appeal condition. However, the appeal type did not influence the participants’ attitudes in the easy-to-process campaign. In addition, the authors found unpleasant feelings to be an underlying mechanism in the effect of interaction between the level of processing fluency and appeal type in the integrated model.

## General Discussion

The present research examined how processing fluency affected participation in a prosocial campaign. In the context of prosocial behavior, the authors found that participants’ unpleasant feelings were generally low in conceptually easy-to-process campaigns, regardless of the benefits these campaigns appealed to. However, difficult-to-process campaigns increased unpleasant feelings. Participants in difficult-to-process campaigns who were manipulated with self-benefit appeal conditions had more unpleasant feelings than those in the social benefit appeal condition. Similarly, the authors found that the participants had less positive attitudes toward the perceptually difficult-to-process campaign after using more effort to process it. Unpleasant feelings mediated this effect on participant attitudes toward the campaign.

These findings contribute to the literature on appeal and processing fluency. First, in this research, the authors used both conceptual and perceptual processing and demonstrated how to increase overall participation in prosocial campaigns. Second, the authors demonstrated that unpleasant feelings mediated the impact of appeal type and processing fluency on prosocial behavior. Previous research found that the means were viewed as more instrumental in fulfilling an accessible goal when the means were associated with difficult processing (Labroo and Kim, 2009). However, the findings of this research showed that the means were not viewed as helpful in accomplishing a goal when the means were associated with difficult processing for those who focused on self-benefits. Third, the findings identified a causal mechanism to explain the effects of appeal type on prosocial behavior through an affective process. Our mediation analysis demonstrated that people motivated by self-benefit decreased their prosocial behavior due to unpleasant feelings while processing a disfluent campaign.

These findings have important practical implications for brands and organizations that use prosocial campaigns. First, the authors demonstrated that processing fluency increases participation or improves attitudes toward prosocial campaigns. Organizations seeking to increase participation in prosocial behavior should avoid making advertisements difficult to read for self-benefit-motivated consumers. Companies design their symbols to influence consumer attitudes toward brands (Pecot et al., 2018). However, if a font or symbol is too complex, it is difficult for consumers to process, especially in long advertisements. Thus, business practitioners promoting prosocial campaigns should avoid using difficult-to-process visual stimuli for consumers who are motivated by self-benefit appeal. Second, our results suggest that improving processing fluency is an effective strategy for companies to improve consumer attitudes toward prosocial campaigns. For example, by increasing the frequency of consumers’ exposure to an advertisement (Janiszewski and Meyvis, 2001) or increasing the probability of recalling the advertisement (Moore et al., 2005), companies can help consumers to understand difficult-to-process campaigns, such as those containing jargon (as in Study 1).

This research revealed the importance of unpleasant feelings in individual responses toward campaigns, which is consistent with previous research. However, we expect that negative feelings have distinct outcomes, and that they should therefore be identified and investigated individually. For example, guilt and shame are both negative emotions that lead people to judge themselves as socially undesirable (Tangney, 1995; Tracy and Robins, 2004), and these are triggered when people conclude that they are not acting in a way that helps them accomplish their own goals (Tracy and Robins, 2004). In particular, when people experience feelings of guilt, they judge their concrete actions negatively (Brown and Weiner, 1984; Blum, 2008). In the example of performing poorly on a test (Lewis, 1971,^ 2000^; Van Vliet, 2009), the guilt-laden person might reflect on specific behaviors (e.g., “I am guilty because I did not study hard last night”), while the shame-laden person would reflect on global selves or capacity as a whole (e.g., “I feel shame because of my lack of intelligence”). Disappointment and regret are other negative feelings also present in the process of individual appraisal, but they differ in their effect on negative emotions from failed experiences (Zeelenberg and Pieters, 2007). Thus, future research should focus on individual negative emotions, which would help clarify the relationship between processing advertisements and intention to participate when individuals are motivated by specific benefit appeals.

In conclusion, the experiments above demonstrated that appeal type shapes motivation to participate in a prosocial campaign based on the level of processing fluency. The findings revealed that, for people motivated with a self-benefit appeal, a difficult-to-process campaign decreases consumer attitudes by increasing their unpleasant feelings. Both conceptual and perceptual fluency interacted with appeal type to influence consumer attitudes toward the campaigns. These findings can help both business practitioners and organizations conduct successful prosocial campaigns.

## Data Availability Statement

The datasets generated for this study are available on request to the corresponding author.

## Ethics Statement

The studies involving human participants were reviewed and approved by Temple University. The patients/participants provided their written informed consent to participate in this study.

## Author Contributions

YeK and YrK jointly designed, analyzed, and wrote up this paper. YeK and YrK undertook all testing and data collection.

## Conflict of Interest

The authors declare that the research was conducted in the absence of any commercial or financial relationships that could be construed as a potential conflict of interest.

## Supplementary Material

The Supplementary Material for this article can be found online at: https://www.frontiersin.org/articles/10.3389/fpsyg.2020.01221/full#supplementary-material.
