# Supplementary material for: The Effects of Processing Fluency in Prosocial Campaigns: Effort for Self-Benefit Produces Unpleasant Feelings
Source: Front Psychol. 2020 Jun 18;11:1221. doi: 10.3389/fpsyg.2020.01221 (PMC7314962; doi:10.3389/fpsyg.2020.01221)
Supplement: Supplementary file 1 [file Data_Sheet_1.docx]

**Appendix**

Table 1. Moderated mediation analysis results for Study 2.

|  | Dependent Variables | | | | | | | | | | | |
| --- | --- | --- | --- | --- | --- | --- | --- | --- | --- | --- | --- | --- |
|  | Regression 1  Unpleasant feelings | | | | |  | Regression 2  Attitudes toward the campaign | | | | | |
|  | *B* | *t* | | *p* | | | | *B* | | | *t* | *p* |
| Intercept | 2.4130 | | 8.9581 | | < 0.0001 | | | 4.9586 | | | 23.5452 | < 0.0001 |
| Appeal type [X] | 0.4758 | | 1.2981 | | 0.1958 | | | −0.1727 | | | −0.8294 | 0.4079 |
| Processing fluency [W] | 0.4270 | | 1.1439 | | 0.2541 | | | |  | |  |  |
| X × W | −1.2946 | | −2.4816 | | 0.0139 | | |  | | |  |  |
| Unpleasant feelings [M] |  | |  | |  | | | 0.0921 | | | 1.6307 | 0.1046 |
|  |  | |  | |  | | |  | | |  |  |
| Regression model | *F*(3, 193) = 2.4362, *p* = .0660  *R*^2^ = 0.0365 | | | | | | |  | | *F*(2, 194) = 1.7316, *p* = 0.1797  *R*^2^ = 0.0175 | | |
